# The Female Threat

**DOI:** 10.1007/s00270-018-1915-2

**Published:** 2018-02-28

**Authors:** Anna-Maria Belli, Meridith Englander

**Affiliations:** 1 Radiology Department, St. George’s University Hospitals, London, UK; 2 Vascular and Interventional Radiology, Albany Medical Center Hospital, Albany, NY USA

It has only been 100 years or less since women earned the right to vote throughout Europe and in the USA. Since that time, women have entered the workforce and joined the professions. Whereas once they were barred from professional medical practice, the percentage of medical school graduates that were women rose from approximately 10% in the 1960s to over 50% in the early part of this century. The number of women physicians is also increasing. This past year, for the first time ever, the province of Quebec [1] reported more female physicians in practice than male. This changing demographic has implications for medical practice as women are needed in all the specialties to ensure equitable availability of services for patients.

According to the 2016 UK Radiology workforce census, 35% of consultant radiologists and 39% of trainees are female, but only 10% of the current consultant IR body is female. At both CIRSE and SIR, only 12% of full members are women. Although precise figures vary between countries and continents, this phenomenon is repeated globally. Interventional Radiology (IR) is under threat, not just by competing specialties, but by its apparent lack of attraction to women. Unless IR is able to reverse this and attract more women, it will be missing out on some of the most talented medical graduates and may have trouble filling all the jobs. In the UK, there is already a crisis of not enough interventional radiologists to meet the need.

In 2009, the Royal College of Physicians of the UK published research into the implications of the rapidly increasing share of female doctors on the medical profession [2]. The two major findings from this report were that women doctors had a far greater preference for flexible working arrangements with scheduled work hours and they preferred specialties offering greater patient interaction. There are many aspects of IR which should appeal to women. It offers patient interaction and longitudinal care and the opportunity to make a real difference to patients’ lives using innovative, minimally invasive procedures. It is constantly progressing and evolving and consequently, never boring. IR is inclusive of almost every body system, and there is the opportunity to develop a subspecialty interest in areas including interventional oncology, vascular disease, women’s health, neurovascular and paediatrics, to name but a few.

So why aren’t women flocking to IR?

Assuming they learn about IR in medical school (and that is an issue in itself; Lee and Lee [3]), the fact that radiation is involved is a big deterrent. This is despite the fact that nowadays occupational radiation exposure to IRs is similar to the natural background dose and most female IRs who continue to work through their pregnancy have foetal radiation doses well below recommended guidelines (Dauer et al. [4], Ghatan et al. [5]). Medical graduates choosing their career need to know these facts, but some of those practising IR inadvertently perpetuate misinformation by excluding or discouraging women who are pregnant from performing fluoroscopically guided interventions. This gives the message that occupational radiation exposure is dangerous. The result is that women’s training is derailed and the pregnant woman is perceived as a burden to her IR colleagues who have to cover the work and on-call responsibilities. Nobody would argue that it is a woman’s choice to avoid radiation exposure during pregnancy, but it should be made clear that this is a choice with two valid alternatives.

Some will prefer to select a specialty offering a different work–life balance, with less emergency work. This is true for both genders and applies to many specialties. There is no doubt that women will and can work hard and long, just look at the number of female obstetricians. Perhaps most of IR has not applied itself to imagining flexible work schedules which allow staff to have more predictable working hours (Deipolyi et al. [6]). This may be a consequence of insufficient numbers of IRs to allow flexibility. Or perhaps, there is no perceived need for change by a currently male-dominated specialty.

The lack of female role models is also a problem. If women do not see other women flourishing in a specialty, they are likely to think it is an unsuitable career choice for them. After all, if it were a great field for women, wouldn’t there be more women? It is imperative that female IRs show themselves and speak up by taking leadership roles in their departments and at the society level. It is also incumbent on our male colleagues to act as allies for women. We need to work together to assure that women in IR are given the same opportunities to succeed as men.

These two authors have had stimulating and satisfying careers in IR, have achieved international recognition, received great support from male and female colleagues alike and have enjoyed successful family lives. We are not exceptional but dedicated to the specialty that we love and to the patients that we treat. And, we are not alone.

Many IR societies throughout the world are awakening to the threat that attracting insufficient numbers of women to IR poses and are encouraging women to get involved. We owe it to our patients that this specialty should continue to thrive and innovate. That can only be done by continuing to inspire and attract the brightest graduates who are increasingly women. The workforce needs to reflect the population, allowing patients’ choice not only in how they are treated, but also by whom they are treated.

If we fail in this, IR will fail too. If we succeed, then we will have a well-balanced, intelligent and expanding workforce with a successful future.
